# Meconium stained liquor and its neonatal outcome

**DOI:** 10.12669/pjms.346.15349

**Published:** 2018

**Authors:** Nadia Mohammad, Taha Jamal, Arjumand Sohaila, Syed Rehan Ali

**Affiliations:** 1 *Dr. Nadia Mohammad, MBBS, FCPS. Senior Instructor Pediatrics, Department of Pediatrics, Aga Khan University Hospital, Karachi, Pakistan*; 2 *Dr. Taha Jamal, MBBS, FCPS. Instructor, Department of Pediatrics, Aga Khan University Hospital, Karachi, Pakistan*; 3 *Dr. Arjumand Sohaila, MBBS, FCPS. Assistant Professor, Department of Pediatrics, Aga Khan University Hospital, Karachi, Pakistan*; 4 *Dr. Syed Rehan Ali, MBBS, DCH, MRCPI, CCST, FRCPCH, Dip HPE. Associate Professor, Indus Hospital, Karachi, Pakistan*

**Keywords:** Meconium aspiration syndrome, Meconium stained amniotic fluid, gestational diabetes

## Abstract

**Objective:**

To determine the maternal factors and neonatal outcome of pregnancy complicated by meconium stained amniotic fluid.

**Methods:**

This one year retrospective study was conducted at the Agha Khan Hospital for Women-Garden Campus, it is a secondary care private teaching hospital. Demographics information included gestational age, gender and birth weight of baby, medical and obstetric complications during pregnancy, mode of delivery, neonatal outcome (Meconium Aspiration Syndrome (MAS) and need for admission in nursery) were recorded on a pre-designed proforma.

**Results:**

In our study the frequency of meconium stained amniotic fluid (MSAF) was 7.85%, out of them 12 % babies developed MAS. There was significant association between grades of meconium and MAS, babies with thick meconium were prone to develop MAS (P = 0.02). Emergency cesarean section was significantly associated with MAS. Gestational diabetes (GDM) and pregnancy induced hypertension (PIH) were the significant factors associated with MAS.

**Conclusion:**

Thick Meconium stained amniotic fluid was associated with low APGAR score, high rate of emergency cesarean section and meconium aspiration syndrome. Anemia during pregnancy, PIH and GDM were important risk factor associated with MAS.

## INTRODUCTION

Meconium stained amniotic fluid (MSAF) is an alarming sign of fetal compromise and associated with a poor perinatal outcome. Incidence of meconium stained amniotic fluid ranges from 7-22%^1^ while meconium aspiration syndrome (MAS) occurs in approximately 5% of all cases of MSAF. MAS contributes to neonatal death in up to 0.05% (i.e. 1 in 2000 of all pregnancies).^2^ Unfortunately Pakistan is number three among those ten countries who contribute two-thirds of the world’s neonatal deaths with an estimated neonatal mortality rate of 49 per 1000 live births.^3^ One such attribute is MSAF, 27.3% of neonatal deaths had a history of or evidence of meconium passage during delivery.^4^ Passage of meconium is not common before 34 weeks of gestation, beyond that period the incidence gradually increases.^5^

Vagal stimulation from umbilical cord compression causing fetal hypoxic stress, resulting in increased peristalsis and relaxation of anal sphincter leading to intrauterine passage of meconium.^6^ Placental insufficiency, maternal hypertension, pre-eclampsia, oligohydramnios or maternal drug abuse (tobacco, cocaine) are predisposing factors of in utero passage of meconium.^7^ Meconium stained neonates are more prone to develop respiratory distress than neonates born with clear fluid. MSAF predisposes perinatal mortality even in women with very low risk for obstetric complications.^5^ MSAF is associated with higher rate of caesarean delivery, instrumental delivery, NICU admission rate, fetal distress, low birth weight and neonatal death.^8^

Therefore identification of maternal factors may help to anticipate the need for neonatal resuscitation in delivery room which eventually helps to improve the perinatal outcome and reduce perinatal mortality and morbidity associated with MSAF. Aim of this study was to determine the maternal factors and neonatal outcome of pregnancy complicated by meconium stained amniotic fluid.

## METHODS

This retrospective study was conducted at the Level-II Nursery of Agha Khan Hospital for Women (AKHW) Garden, from January 2013 – December 2013. AKHW-Garden is a secondary care and a private teaching hospital; caters to a class of patients that belong to lower and middle class social strata. Patients with gestational age >37 weeks, cephalic presentation, who presented with meconium stained liquor after spontaneous or artificial rupture of membranes during labour were enrolled and their records were reviewed, while all neonates with congenital anomalies were excluded. The study was carried out after obtaining approval from the Aga Khan University Hospital ethical review committee. As this was retrospective study there was no need to take subject consent.

A pre-designed proforma was filled by reviewing of clinical notes which entailed information about basic demographic information gestational age, birth weight, gender of baby, booking status of mother, maternal clinical characteristics, medical and obstetric complications during pregnancy, premature rupture of membrane (PROM), grades of meconium, mode of delivery, neonatal outcome (APGAR score, type of resuscitation, meconium aspiration syndrome and need for admission in nursery).

Gestational age (recorded as completed weeks) was calculated from maternal last menstrual period (LMP) to date of delivery and was categorized as preterm less than 37 weeks gestation and term 37 weeks or above. In routine practice, birth anthropometries were measured by staff nurse in labor room or operation theatre by using standardized equipment (Laiqa by Italy). Weight was measured without clothes using standard weighing balance in kilogram (kg). The calibration of the weighing scale was checked regularly before each measurement in order to avoid error, baby was placed on the weighing scale ensuring that entire body was on the scale, then weight was adjusted until the balance beam was centered, after that reading was documented and plotted on specific WHO growth charts (Fenton growth chart) percentiles were noted and categorized as low birth weight (LBW) if weight was less than 2.5kg, appropriate for gestational age (AGA) was more than 2.5kg.

Information regarding obstetrics complications such as anemia, pregnancy induced hypertension (PIH) and gestational diabetes (GDM), maternal age parity and antenatal care (ANC) were also obtained.

Consistency of meconium was categorized into thick and thin. Thick greenish meconium with particulate matter in amniotic fluid was considerd as thick meconium while thin meconium was defined as light greenish staining of amniotic fluid.^9^ Comparisons were made between grades of meconium to identify any significant difference between the two groups in association with neonatal outcomes.

Cardiac tocography (CTG) and mode of delivery (cesarean section C/S, Vaginal delivery with and without instrumental delivery) were also recoded; CTG was categorized as reassuring and non-reassuring. Neonatal outcome included APGAR score at one minute, Low birth weight, general condition (symptomatic or remained asymptomatic), need for admission in nursery and development of meconium aspiration syndrome (MAS) were also recorded. Meconium aspiration syndrome was defined as presence of meconium stained skin, umbilical cord or meconium in the trachea at birth, followed subsequently by signs and symptoms of MAS (i.e. tachypnea, dyspnea, retraction, grunting or cyanosis).^9^

### Plan of Analysis

Data was analyzed by SPSS 20. Results for continuous variables like gestation age, weight were expressed as mean ± SD. Frequency and percentage was calculated for categorical variables like booking status, mode of delivery, gender, illness during pregnancy, parity, APGAR score, CTG and neonatal outcomes. Confounding variable such as previous C-section was adjusted by stratification of data into categories. The chi square test was applied between grades of meconium and neonatal outcome at 95% confidence interval and the p-value <0.05 was considered to be statistically significant and P-value also calculated based on person Chi square test between MSAF and MAS.

## RESULTS

During the study period the total number of deliveries was 1898 and 149 (7.85%) cases had meconium stained amniotic fluid. Out of 149 cases of MSAF, 18 (12%) neonates developed meconium aspiration syndrome. The mean birth weight of MSAF patients was 3.04 kg (± 0.38, range: 1.96-4.6), LBW was seen in 6% of MSAF babies. Among 149 women with MSAF, 99(66.4%) women had cesarean section while 50 (33.6%) delivered through spontaneous vaginal delivery. Table-I shows neonatal and maternal demographic characteristic in patient with MSAF.

**Table-I:** Neonatal and maternal demographic Characteristics (n=149).

Demographic characteristics	Number (%)
Gestational age (week)	39.1(±1.09) (Mean±SD)
Birth weight (kg)	3.04 (±0.38) (Mean±SD)
Maternal Age (year)	26.7(±4.4) (Mean±SD)
*Gender:* M	66(44.3%)
F	83(55.7%)
Booking status: Booked	131(87.9%)
Unbooked	18(12.1%)
*Parity:* Primipara	76(51%)
Multipara	73(49%)
Pregnancy induced hypertension	08(5.4%)
Gestational Diabetes	18(12.1%)
Anemia in pregnancy	38(25.5%)
*Grades of MSAF:* Thin	114(76.5%)
Thick	35(23.4%)
IUGR	07(4.7%)
PROM	20(13.4%)
*Rupture of membrane:* Spontaneous	48(32.2%)
Artificial	73(49%)
Non-reassuring CTG	43(28.8%)

Neonatal outcome and mode of delivery in relation to the grades of meconium is shown in Table-II. Low APGAR score, Non-reassuring CTG, MAS, and emergency Cesarean section were significantly associated with thick meconium stained liquor. There were 114 (76.5%) cases of thin meconium; out of them 83.3% babies remained asymptomatic, which was not statistically significant. PIH and GDM were important risk factors associated with MAS (P-value <0.001), whereas no significant association was found with parity. Table-III shows maternal risk factors in patient with MSAF in relation to the MAS group.

**Table-II:** Neonatal outcome and mode of delivery in relation to grades of meconium.

Neonatal Outcome	Thin MSAF (n=114)	Thick (n=35)	p-value
Remained Asymptomatic	95(83.3)	25(71.4)	0.87
Low APGAR<7	05(4.3)	09(25.7)	<0.001*
LBW	07(6.1)	02(5.7)	0.91
IUGR	07(6.1)	00	0.13
Non-Reassuring CTG	26(22.8)	18(51.4)	0.001*
Immediate resuscitation	07(6.1)	03(8.5)	0.61
endotracheal suctioning	07(6.1)	06(17)	0.044*
Nursery admission	15(13.1)	10(28.5)	0.001*
MAS	10(8.7)	08(22.8)	0.025*
Emergency C/S	68(59.6)	25(71.4)	0.003*

* stastically significant.

**Table-III:** Maternal factors associated with meconium stained amniotic fluid and meconium aspiration syndrome.

Maternal factor	MSAF # (%) (n=149)	MAS # (%) (n=18)	p-value
Antenatal care Booked	131(87.9)	01(5.5)	
Unbooked	18(12.1)	17(94.5)	<0.001*
***Parity***			
Primipara	76(51)	11(61)	0.36
Multipara	73(49)	07(39)	
Anemia in pregnancy	38(25.5)	17(94.5)	0.001*
PIH	08(5.4)	04(22.2)	<0.001*
GDM	18(12.1)	10(55.5)	<0.001*
PROM	20(13.4)	6(33.3)	0.008*
***MOD***			
SVD	50 (33.5)	4 (22.2)	0.27
C-Section	99 (66.4)	14 (77.7)	

* Significant difference between proportions using Pearson Chi- squared test at 0.05 level of significance.

Out of 18 babies with MAS two were referred due to requirement of mechanical ventilation and none of them was expired. Only 1.3% of the babies needed mechanical ventilation, stating that the worrisome outcome can be prevented with early screening, prompt recognition, quick and proper management of the babies with meconium stained amniotic fluid.

## DISCUSSION

Meconium stained amniotic fluid, a troublesome situation both for obstetrician and pediatrician, it is associated with high rates of caesarean section, perinatal morbidity and mortality. The rate of meconium-stained amniotic fluid varies from 12 to 20%.^10^ It is higher in underdeveloped countries. In our study we found incidence of 7.85% for MSAF. As the gestational age increases, the incidence of MSAF also increases which was very obvious in this study. All of the cases had gestational ages of more than 37 weeks, similar finding were observed in study by Desai et al.^11^

We found 76.5% of cases having thin meconium stained fluid and 23.5% with thick meconium, these findings consistent with the study of Hanoudi,^12^ but in contrast with study by Khazardoost et al, where 10.6% patients had thin meconium while 89.4% patients had thick meconium.^13^

The CTG changes are the reflection of mechanical stress on the foetus, we found 71.2% patients with MSAF had reassuring CTG, while the remaining (28.8%) had non-reassuring CTG, which predominantly associated with thick meconium (p-value <0.001) while Desai et al did not find any association with CTG pattern in thick meconium.^11^

It has been observed that obstetricians are more aggressive while managing labour with MSAF leading to high cesarean section rate, which was stastically significant in our study, more number of women with thick meconium underwent cesarean section as compared to thin meconium (60% vs 71%p-value 0.003). These findings are comparable with the study by Kumar S et al,^14^ in which cesarean delivery was higher 72% in thin meconium in contrast to thick meconium 21%. A possible explanation could be due to thick meconium occurring later in second stage.

Thick meconium led to more nursery admissions when compared to their counterpart (p-value 0.001), this was also the case with Desai study.^11^ Most of the babies in both groups (thin and thick) do not usually require resuscitation and remain asymptomatic and was not found stastically significant. Endo-tracheal suction was needed in cases of thick meconium (p-value 0.04).

MAS is well known complication of MSAF with incidence varies from 1-6.8% in babies born with MSAF,^15^ in our study it was found in 0.94% of total deliveries and 12% of MSAF cases and was highly observed in cases with thick meconium than thin meconium (p-value 0.02). MAS increases neonatal intensive care unit admissions, long term morbidity and mortality; strategies to prevent MAS need to be practical, safe, effective, and based on risk assessment.

MAS was found more in babies born to unbooked mothers (p-value <0.001) signifying the importance of antenatal care. In present study, maternal risk factors significantly associated with MAS were anemia in pregnancy, gestational diabetes, PIH and premature rupture of membranes while none of the mothers had pre-eclampsia.

Anemia in pregnancy and PIH were found to be associated with high incidence of MAS (p-value <0.03 and <0.001). Ashtekar in India also found maternal anemia and PIH one of the antenatal risk factors for MAS.^16^

In our study gestational diabetes was one of the risk factors associated with MAS (p-value<0.001), while Aviram A et al could not find any significant association between gestational diabetes and MAS.^17^

## CONCLUSION

Thick meconium stained amniotic fluid has a major impact on both mode of delivery and neonatal outcome as compared to the other counterpart. GDM and PIH were risk factors associated with meconium aspiration syndrome. Therefore presence of thick meconium needs close monitoring, early and timely obstetrical intervention and appropriate post natal care, in order to minimize meconium related complications and improve fetal outcome.

### Authors’ Contribution

**NM:** Conceived, designed and did statistical analysis & editing of manuscript.

**TJ & AS:** Did data collection and manuscript writing.

**RA:** Did review and final approval of manuscript.
